# Multistate Outbreak of *Salmonella* Chester Infections Associated with Frozen Meals — 18 States, 2010

**Published:** 2013-12-06

**Authors:** Joshua Rounds, Julie Schlegel, Tom Lane, Jeffrey Higa, Bonnie Kissler, Wright Culpepper, Ian Williams, Leslie Hausman

**Affiliations:** Minnesota Dept of Health; South Carolina Dept of Health and Environmental Control; Tennessee Dept of Health; California Dept of Public Health; Food Safety and Inspection Svc, US Dept of Agriculture; Div of Foodborne, Waterborne, and Environmental Diseases, National Center for Emerging and Zoonotic Infectious Diseases, CDC

On May 24, 2010, a cluster of 17 human *Salmonella enterica* serotype Chester clinical isolates with indistinguishable pulsed-field gel electrophoresis (PFGE) patterns was reported to PulseNet, the national molecular subtyping network for foodborne disease surveillance. This PFGE pattern had not been reported previously. Subsequently, CDC conducted an investigation that identified 44 ill persons in 18 states during May 24–June 19, 2010. In a multistate case-control study, consumption of a brand A frozen meal was associated with illness (matched odds ratio [mOR] = 30.7; 95% confidence interval [CI] = 6.4–∞). On June 17, 2010, the manufacturer (company A) voluntarily recalled its brand A cheesy chicken and rice frozen meals. The outbreak strain of *Salmonella* Chester was isolated from eight unopened samples. A root cause analysis conducted by company A identified chicken as a possible contaminated ingredient. Many frozen meals are not “heat and serve” items but rather are “not-ready-to-eat” (NRTE) products that require full cooking before consumption because they might include ingredients that have not gone through a pathogen kill-step process. Because *Salmonella* and other pathogens can survive in NRTE products, such products must be fully cooked before eating and clearly labeled with instructions for safe handling and cooking.

## Epidemiologic Investigation

**FIGURE 1:**
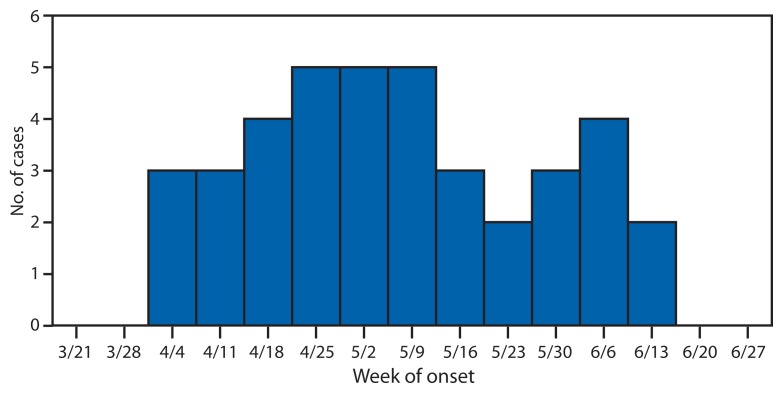
Number of confirmed cases (N = 44) of infection with the outbreak strain of *Salmonella* Chester, by week of illness onset^*^ — 18 states, April 4–June 19, 2010 ^*^ Week of illness onset was not reported for five of the 44 confirmed cases.

**FIGURE 2:**
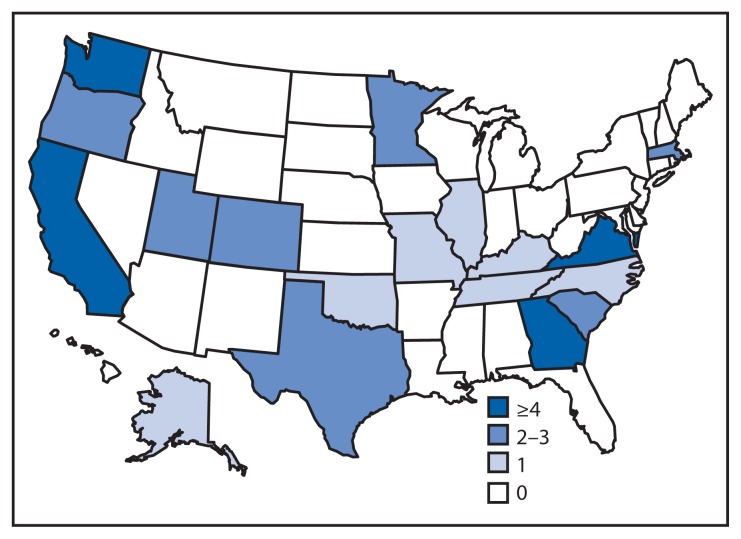
Number of confirmed cases (N = 44) of *Salmonella* Chester infection in outbreak associated with frozen meals^*^ — 18 states, April 4–June 19, 2010 ^*^ The outbreak strain was identified by pulsed-field gel electrophoresis pattern.

For this investigation, a case was defined as a laboratory-confirmed infection with the outbreak strain PFGE pattern JCPX01.0060 of *Salmonella enterica* serotype Chester and illness onset during April 4–June 19, 2010 (Figure 1). A total of 44 cases from 18 states were identified (Figure 2). The median age of patients was 36 years (range: <1–88 years), 30 (73%) of 41 patients were aged >19 years, and 21 (54%) of 39 were female. Among 43 patients with available information, 16 (37%) were hospitalized; no deaths were reported.

During June 4–11, 2010, ill persons were interviewed using a structured questionnaire to assess exposure to approximately 300 food and other items; these hypothesis-generating interviews revealed that six of 11 persons with infection reported eating brand A frozen meals before illness onset. A matched case-control study was initiated on June 14, 2010. Case-patients aged >2 years were enrolled. Controls were recruited from well persons among neighbors of case-patients identified by reverse-digit dialing and were matched by age group (<40 and ≥40 years). The questionnaire included questions on the consumption of items commonly reported during hypothesis generation (i.e., frozen meals, cereal, chicken, and lettuce). Case-patients were asked about exposures during the week before illness onset, and controls were asked about exposures in the week before their interview. Totals of 11 case-patients and 22 controls were enrolled from seven participating states.

Consuming a brand A frozen meal was significantly associated with illness. All 11 of the case-patients reported eating a frozen meal, compared with three (14%) of the 22 control subjects (mOR = 24.3) (Table). The same case-patients reported eating a brand A frozen meal, whereas none of the three controls who reported eating a frozen meal ate a brand A meal (mOR = 30.7). Cheesy chicken and rice was the most commonly consumed brand A frozen meal, reported by eight (73%) of the 11 case-patients, followed by three (27%) consuming sweet and sour chicken. No other food item was associated with illness (Table).

After completing the case-control study, patients were interviewed using a standard questionnaire to further explore the types of brand A frozen meals potentially linked with illness. Among the 31 patients from whom information was collected, 25 (81%) reported consuming a frozen brand A meal during the week before illness onset. A total of 21 (84%) of 25 reported eating a brand A cheesy chicken and rice meal. In addition, patients were asked questions regarding how they cooked their frozen meals. Twenty-one (84%) of 25 reported cooking their frozen meal in a microwave, whereas five (20%) cooked their frozen meal in a conventional oven. A total of 22 (88%) let their meal stand for the time recommended in the cooking instructions before eating, and six (25%) of 24 cooked more than one meal at a time using the same method (microwave or oven).

## Control Measures

On June 17, 2010, CDC informed company A of the association between brand A cheesy chicken and rice frozen meals and the outbreak of *Salmonella* Chester infections. That day, the U.S. Department of Agriculture’s Food Safety and Inspection Service (USDA-FSIS) convened its Recall Committee (1), and company A announced a recall of all brand A cheesy chicken and rice frozen meals, regardless of production date. This recall was conducted based on the strength of the epidemiologic data, and was done before the strain was isolated from brand A cheesy chicken and rice frozen meals.

## Environmental Investigation

The outbreak strain was later isolated from eight unopened brand A cheesy chicken and rice frozen meals with three production dates ranging from July 14, 2009 to March 12, 2010. Brand A cheesy chicken and rice frozen meals contained a cooked chicken product, raw broccoli, partially cooked rice, and cheese. The cooked chicken was produced by company B; USDA-FSIS reviewed company B’s hazard analysis and critical control point plan and sanitation records and did not find any deficiencies.

During July 7–August 9, 2010, USDA-FSIS and the Food and Drug Administration (FDA) Center for Food Safety and Applied Nutrition and Office of Regulatory Affairs conducted a comprehensive food safety assessment at company A, where the cheesy chicken and rice meal was produced, and did not identify any significant food safety issues. FDA conducted a traceback investigation into the sources of broccoli, but did not identify any common suppliers. Company A conducted a root cause analysis to identify common sources for ingredients used for the three production dates where the outbreak strain had been isolated. This extensive review identified a single poultry farm as a common supplier of chicken to a chicken cooking facility, company B. The three production dates of interest suggested that cooked chicken might have been the contaminated ingredient.

### Editorial Note

Outbreaks of *Salmonella* and Shiga toxin–producing *E. coli* infections associated with consuming frozen NRTE entrées have been previously reported (2–6). A common feature among these outbreaks is the consumer’s misconception that the microwave process is for palatability and reheating, and not a critical control point to ensure raw and uncooked ingredients in NRTE products reach a sufficient temperature to render them safe from microbial hazards. Although safe handling instructions must be displayed in a prominent manner using terms that are easily understood such as uncooked, raw, or NRTE (7), a lack of clear cooking instructions on food product packaging, combined with consumers’ limited knowledge of the wattage on their microwave ovens, appeared to be important factors contributing to the previous outbreaks.

A majority (84%) of U.S. residents report using their microwave oven to prepare packaged products. However, a survey conducted in 2010 found that only 69% followed all the cooking instructions (8). Another survey found that only 26% of participants reported they knew their microwave wattage (9).

What is already known on this topic?

*Salmonella* commonly causes foodborne illness, and ingredient-driven outbreaks are difficult to detect. Not-ready-to-eat (NRTE) microwave products contain raw, uncooked ingredients and can contain pathogens that cause foodborne illnesses.

What is added by this report?

In May 2010, CDC identified a cluster of 17 human *Salmonella enterica* serotype Chester clinical isolates with indistinguishable pulsed-field gel electrophoresis patterns; the pattern had not been reported previously. The investigation identified 44 ill persons in 18 states. The potential source was chicken in an NRTE cheesy chicken and rice frozen meal.

What are the implications for public health practice?

Food manufacturers should place step-by-step, easy to follow, product-specific cooking instructions on all NRTE frozen microwavable products. Consumers should know the wattage of their microwave, and carefully read and follow instructions printed on the packaging for preparing NRTE frozen microwave entrées, including microwaving and allowing the product to stand for the recommended time before consuming.

In this outbreak, brand A cheesy chicken and rice packaging provided clearly marked cooking instructions for both microwave and conventional ovens; labeling for safe handling was displayed on both sides of the packaging, stating that the product must be “cooked thoroughly.” However, not all of the persons with *Salmonella* Chester infection who were interviewed reported allowing their meal to stand for the time recommended in the cooking instructions before eating; microwave standing time is part of the cooking process.

Although no definitive cause was identified, company A’s investigation suggested that cooked chicken in the frozen meal might have been contaminated. Company A has since implemented changes in its frozen foods Food Safety and Quality Programs. The company is now testing finished products and selected raw materials for various pathogens and partnering with suppliers to initiate more robust testing of lots dedicated for frozen meals. In addition, company A has developed internal methods to improve its processes at manufacturing establishments and has added consumer handling of frozen meals to its hazard analysis (10).

This outbreak highlights the need for consumers to thoroughly cook frozen foods that are NRTE because they contain raw ingredients. Manufacturers should clearly label products as NRTE and as containing raw ingredients. Food manufacturers should place step-by-step, easy to follow, product-specific cooking instructions on all NRTE frozen microwavable products. These instructions should be validated to account for variability in microwave wattage. Microwave oven manufacturers should clearly indicate the oven wattage on the front of the appliance. Consumers should know the wattage of their microwave and carefully read and follow instructions printed on packaging on how to properly heat and prepare NRTE frozen microwave entrées. Consumers should not only follow instructions for microwaving but should also allow the product to stand for the recommended time before consuming. Additionally, a food thermometer should be used to ensure that entrees are fully cooked and that all ingredients reach at least 165°F (74°C).

## Figures and Tables

**TABLE:** Comparison between case-patients with *Salmonella* Chester infection and control subjects, by selected food exposures — 18 states, 2010

Food exposure	Case-patients (n = 11)		Controls (n = 22)		Matched odds rato	(95% CI)
	
	No.	(%)	No.	(%)		
**Frozen meal**	11	(100)	3	(14)	24.3	(4.9–∞)
**Brand A frozen meals**	11	(100)	0	—	30.7	(6.4–∞)
Cheesy chicken and rice	8	(73)	0	—	22.1	(4.4–∞)
Sweet and sour chicken	3	(27)	0	—	7.7	(1.2–∞)
Fettuccini with chicken and broccoli	2	(18)	0	—	4.8	(0.6–∞)
Pot pie	2	(18)	0	—	4.8	(0.6–∞)
Fried chicken and gravy	2	(18)	0	—	4.8	(0.6–∞)
Turkey breast with stuffing	2	(18)	0	—	4.8	(0.6–∞)
Beef tips in mushroom sauce	2	(18)	0	—	4.8	(0.6–∞)
Beef and broccoli	2	(18)	0	—	4.8	(0.6–∞)
Meat lasagna	2	(18)	0	—	4.8	(0.6–∞)
**Pre-cut chicken parts**	5	(45)	9	(41)	5.8	(0.6–295.4)
**Boxed cereal**	9	(82)	13	(59)	2.8	(0.5–30.9)
**Bagged lettuce**	3	(27)	9	(41)	1.3	(0.2–10.5)
**Butter**	3	(27)	13	(59)	1.0	(0.01–97.9)
**Peanut butter**	3	(27)	10	(45)	0.9	(0.1–7.3)
**Bananas**	3	(27)	13	(59)	0.5	(0.04–5.4)

**Abbreviation:** CI = confidence interval.

## References

[1] US Department of Agriculturem, Food Safety and Inspection Service (2013). FSIS food recalls.

[2] Smith K, Medus C, Meyer S (2008). Outbreaks of salmonellosis in Minnesota (1998 through 2006) associated with frozen, microwaveable, breaded, stuffed chicken products. J Food Prot.

[3] CDC (2008). Multistate outbreak of *Salmonella* infections associated with frozen pot pies—United States, 2007. MMWR.

[4] Gessner BD, Beller M (1994). Protective effect of conventional cooking versus use of microwave ovens in an outbreak of salmonellosis. Am J Epidemiol.

[5] Evans MR, Parry SM, Ribeiro CD (1995). *Salmonella* outbreak from microwave cooked food. Epidemiol Infect.

[6] CDC (2013). Multistate outbreak of Shiga toxin-producing *Escherichia coli* O121 infections linked to Farm Rich brand frozen food products.

[7] US Department of Agriculture, Food Safety and Inspection Service (2006). FSIS notice 75-06: verification instructions for changes in label requirements for uncooked and raw, frozen, breaded, boneless poultry products.

[8] International Food Information Council Foundation (2010). 2010 food & health survey: consumer attitudes toward food safety, nutrition, & health.

[9] Lando A, Carlton E, Verrill L 2010 food safety survey microwave use questions.

[10] Menke-Schaenzer J Marie Callender’s Cheesy Chicken and Rice *Salmonella* Chester outbreak.

